# Effects of enhanced insect feeding on the faecal microbiota and transcriptome of a family of captive common marmosets (*Callithrix jacchus*)

**DOI:** 10.1371/journal.pone.0279380

**Published:** 2022-12-22

**Authors:** Yumiko Yamazaki, Shigeharu Moriya, Shinpei Kawarai, Hidetoshi Morita, Takefumi Kikusui, Atsushi Iriki

**Affiliations:** 1 Laboratory for Symbolic Cognitive Development, RIKEN Center for Biosystems Dynamics Research, Kobe, Hyogo, Japan; 2 Photonics Control Technology Team, RIKEN Center for Advanced Photonics, Numazu, Shizuoka, Japan; 3 Laboratory of Small Animal Clinics, Veterinary Teaching Hospital, Azabu University, Sagamihara, Kanagawa, Japan; 4 Graduate School of Environmental and Life Science, Okayama University, Okayama, Okayama, Japan; 5 Companion Animal Research, School of Veterinary Medicine, Azabu University, Sagamihara, Kanagawa, Japan; ICMR-Regional Medical Research Centre Bhubaneswar, INDIA

## Abstract

Common marmosets have been widely used in biomedical research for years. Nutritional control is an important factor in managing their health, and insect intake would be beneficial for that purpose because common marmosets frequently feed on insects in natural habitats. Here, we examined the effect of enhanced insect feeding on the gut by analysing the faecal microbiota and transcripts of captive marmosets. A family consisting of six marmosets was divided into two groups. During the seven-day intervention period, one group (the insect feeding group, or Group IF) was fed one cricket and one giant mealworm per marmoset per day, while the other (the control group, or Group C) was not fed these insects. RNA was extracted from faecal samples to evaluate the ecology and transcripts of the microbiota, which were then compared among time points before (*Pre*), immediately after (*Post*), and two weeks after the intervention (*Follow_up*) using total RNA sequencing. The gut microbiota of marmosets showed *Firmicutes*, *Actinobacteria*, *Bacteroidetes*, and *Proteobacteria* as dominant phyla. Linear discriminant analysis showed differential characteristics of microbiota with and without insect feeding treatment. Further analysis of differentially expressed genes revealed increases and decreases in *Bacteroidetes* and *Firmicutes*, respectively, corresponding to the availability of insects under both *Post* and *Follow_up* conditions. Significant changes specific to insect feeding were also detected within the transcriptome, some of which were synchronized with the fluctuations in the microbiota, suggesting a functional correlation or interaction between the two. The rapid changes in the microbiota and transcripts may be achieved by the microbiota community originally developed in the wild through marmosets’ feeding ecology. The results were informative for identifying the physiological impact of insect feeding to produce a better food regimen and for detecting transcripts that are currently unidentifiable.

## Introduction

Many primate species use insects as food resources [e.g., 1–3]. One of the reasons is the various nutritional components present in insects, such as energy, protein, fat, vitamins, and minerals [2, 4]. Supporting the long history of insectivory, primates retain genes encoding enzymes that degrade insect exoskeletons by digesting a polysaccharide, chitin [5, 6]. Thus, they have developed a physiological system that is adapted for insect eating, even if it becomes only complimentary or supplemental to their diets [5].

Common marmosets (*Callithrix jacchus*), a species of New World monkey, feed on a variety of food items, such as insects, fruits, and small animals [7, 8], while they are known to maintain highly exudativorous (i.e., highly dependent on tree exudates, such as gum) diets and morphologies [9]. Among them, insects are important nutritional resources because they account for 30–70% of their diet [10]. These marmosets eat various insects, such as grasshoppers, crickets, cicadas, and cockroaches [7]. Consistent with the observations, the expression of a gene encoding an enzyme that digests insect exoskeletons, CHIA, is the highest in their stomach [6]. Although insects seem to have important roles in maintaining bodily conditions, the unique effect of insects has not been well clarified. In the present study, we aimed to determine the effect of insect feeding on microbiota and transcript levels in captive marmosets by analysing faecal samples.

The gut microbiota of common marmosets has been documented in both captive and wild groups, with and without diseases [e.g., 11–20]. Studies revealed that the phyla *Actinobacteria*, *Bacteroidetes*, *Firmicutes*, *Fusobacteria*, and *Proteobacteria* are abundant in individuals without diseases [see 17 for a review], although high variability has been noted among facilities. The gut microbiome of primates is modulated by various factors, including diets [13, 21, 22], housing conditions [23], pairing [24], aging [15], and diseases [11, 25]. Among them, diet is influential even for only a short period of time. In human subjects, the microbiota was reported to substantially modify microbiota diversity after a change in diet for short periods of time, even from 24 hours after reaching to the gut [21, 26–28]. Thus, we hypothesized that insect feeding would exert a substantial effect on the microbiome of marmosets even in a short period, because they should have developed a physiological system suitable for insect feeding in their evolutional history.

Another consideration regarding the use of insects as nutritional resources for common marmosets is "marmoset wasting syndrome” (MWS, or wasting marmoset syndrome, WMS) that has been observed in captive animals in many facilities [29]. MWS is a well-known health problem endemic to captive marmoset colonies and has been documented for several decades [30, 31], although breeding methods for captive marmosets have been well established [10, 32, 33]. The syndrome consists of various symptoms, but diarrhoea, anorexia, and anaemia are frequently observed [34, 35]. Several causes have been suggested to explain the variable symptoms, and malnutrition is thought to be one of the important factors for the aetiology of MWS [29, 30]. Thus, we hoped to observe a positive effect on the microbial ecology of marmosets through insect feeding as a nutritional mimic of the wild habitat.

In the present study, we evaluated the effects of enhanced insect feeding for seven days on the gut microbiota and transcript levels by comparing groups with and without insect feeding. The main advantage of analysing both microbiota and transcripts simultaneously is to understand functional characteristics that would be attributable to ecological changes in the marmoset gut microbiota. The weekly weight and daily faecal scores were recorded to monitor the general health status throughout the experimental period. RNA was extracted from faecal samples collected at different time points (before (*Pre*), immediately after (*Post*), and two weeks after (*Follow_up*) the experimental intervention), and DNA was then sequenced and annotated with a database for taxonomic identification. We employed total RNA sequencing (total RNA-seq) as the analytical method to describe the activities of the whole microbial community in marmoset guts under our experimental conditions [36]. The total RNA-seq approach has been used in various studies [37–42] to obtain information from all domains of microbial inhabitants, including eukaryotes, archaea, and bacteria, without a strong PCR bias. Additionally, it can describe the gene expression patterns among samples, similar to standard transcriptome analyses, using short-read alignment tools [43–45].

## Materials and methods

### Subjects

Six healthy adult common marmosets (*Callithrix jacchus*) from a family consisting of one mother (9 y) and five offspring (one male and four females, aged 3–4 y) were used in this study. The mean weight was 460 g, with a range from 374 g to 499 g. The mother was obtained from a company (Clea, Tokyo, Japan), and the offspring were laboratory born and raised by their own parents. They were living in a cage (w 70 x d 70 x h 180 cm) vertically separated by a metal mesh plate to prevent fighting; thus, they were physically separated but visually, acoustically, and olfactorily accessible to each other. The cage was placed in a breeding room on a 12-hour light-dark cycle and maintained at a temperature of 28°C and 50% humidity. The marmosets were divided into two groups that differed in terms of the amount of insect intake per week, as described below. After the study finished, the animals were not sacrificed, as the study did not include examination of postmortem specimens.

### Diets

The marmosets were fed commercially available pelleted foods (CMS-1M, Clea, Tokyo, Japan; SPS, Oriental Yeast, Tokyo, Japan) daily ad libitum in the morning and vegetables and fruits in the afternoon, in addition to a variety of food items such as yogurt, boiled eggs, acacia gum, cottage cheese, and small dried sardines. Different probiotic preparations (*Bifidobacterium bifidum* (Biofermin), Biofermin Seiyaku, Hyogo, Japan; *Bifidobacterium animalis* subsp. *lactis* (LKM512), Meito, Tokyo, Japan; *Bifidobacterium longum* and *Bifidobacterium infantis* (LAC-B), Kowa, Aichi, Japan) were added to the meals or administered orally (1/2 to 1 tablet per animal) when the animals presented softened faeces or diarrhoea, including in the experimental periods. Until the beginning of the current study, frozen house crickets (*Acheta domestica*), which were defrosted at the time of feeding, were given to all animals in the colony once per week (usually on Wednesday).

For the insect feeding treatment, we used two different species, a house cricket (Tsukiyono farm, Gunma, Japan) and a giant mealworm (*Zophobas atratus*, Sagaraya, Kumamoto, Japan), which were commercially available and were kept frozen when they were delivered to the laboratory. They were brought back to room temperature to thaw just before feeding. These species have been reported to have similar amounts of protein, while the giant mealworm is much fattier than the cricket with higher calories [46]. Additionally, three crickets and three mealworms were used for microbiota and transcript analyses of these dietary components.

### Procedures

#### Experimental design

The family was divided into two experimental groups, each with three subjects. One group (Group IF, consisting of three offspring females) was fed one cricket and one giant mealworm per day for seven continuous days. The other group (Group C, consisting of the mother, one offspring female, and one offspring male) was fed one cricket per week in the middle of the week, which was the regular food regimen in our colony. These insects were fed manually by the caretakers to each subject during the daytime.

Three points of faecal sampling were established in the study: *Pre*, *Post*, and *Follow_up*. *Pre* samples were collected one week before starting the insect feeding period in the experimental group. *Post* samples were collected the day after the end of insect feeding and *Follow_up* samples were collected two weeks after the insect feeding treatment.

#### Sample preservation

Samples were collected from the clean stainless floor of the breeding cages within 30 minutes of defecation early in the morning (8:30–10:30 AM) when the marmosets usually defecated frequently to analyse the microbiota and transcript levels in the faeces from marmosets. Three pieces of faeces each were collected from the experimental and control groups, without identifying the individuals, and the faeces were immediately placed in 10 ml of RNAlater (Thermo Fischer Scientific, Waltham, MA, US) and manually mixed well with a sterile spatula to homogenize them in the liquid. Using the same procedure, we collected samples three times from each group at each sampling point to prepare three tubes consisting of three faecal pieces in 10 ml of RNAlater. The tubes were allowed to stand for 24 hours at room temperature. Then, they were stored at -80°C in a refrigerator until cDNA construction.

#### RNA extraction, sequencing, and taxonomic annotation

Faecal RNA was purified by using the RNeasy PowerMicrobiota kit (Qiagen, Hilden, Germany). The kit was operated with an automatic system, QIAcube (Qiagen, Hilden, Germany), according to the protocol (RNA_RNeasyPowerMicrobiota_StoolOrBiosolid_IRTwithDNAse_V1.qpf) provided by the manufacturer. The concentration of RNA was measured with a Qubit 2.0 Fluorometer (Thermo Fischer Scientific, Waltham, MA, USA). For library construction, 10 ng of obtained RNA was processed using the SMARTer Stranded RNA-seq kit for Illumina (Takara Bio Inc., Shiga, Japan) according to the manufacturer’s instructions. After the RNA levels were evaluated using qPCR with the KAPA Library Quantification kit (KAPA Biosystems, Wilmington, MA, USA), the libraries were loaded onto an Illumina MiSeq sequencer and then sequenced using MiSeq Reagent kits v2 500 cycles (Illumina, San Diego, CA, USA) to obtain 250 bp paired-end reads. The nucleotide sequence data are available in the data base (see Data Availability section).

We used a mapping-based total RNA-seq pipelines [36] to analyse both rRNA and mRNA profiles to identify the taxonomy of the microbiota and to search for their functions.

The obtained raw paired-end reads were trimmed by using Trimmomatic-0.35 [47] with seed mismatch settings: palindrome clip: simple clip threshold = 5:30:7, minimum read length of 100 bp, head crop of 6 bp and a specification to remove SMARTer kit-specific adaptor sequences. Then, trimmed paired-end reads were directly mapped to the SSU rRNA database SILVA release 128 rep-set data with 99% identity by Bowtie2 [44] with local mode default condition as a “best-hit” analysis. The data were transformed to BAM format for expression analysis. Mapped reads were counted by using eXpress [48] to obtain counting data against the SSU rRNA sequence database. Count data were combined with taxonomy data provided from SILVA release 128 (taxonomy all, 99% identity, taxonomy_7_levels.txt) by R [49].

To analyse the metatranscriptome, paired-end reads were assembled by using the Trinity v2.4.0 program package [50] with paired-end mode default settings. Open reading frames (ORFs) and the encoded protein sequences were predicted using Transdecoder. LongORF script in the TransDecoder v.3.0.0 program package (https://transdecoder.github.io/). The ORF data (longest_orfs.cds) were used as the reference database for read mapping. Mapping was performed as described above for SSU rRNA analysis. Functional annotation of the identified ORFs was conducted with the Trinotate-3.0.1 program package [51] (https://trinotate.github.io/). The obtained functional annotations were combined with read count data using R [49].

The obtained read count data were normalized according to Love et al. [52] using the TCC package in R. Additionally, SSU rRNA reads or ORFs with less than 10 mapped reads in total from all samples in the original count data were excluded using an in-house script of R.

#### Data analysis

We analysed the data considering two types of biological fluctuations identified in the samples: fluctuations related to the intervention and those related to daily activities (thus experimentally uncontrolled). For the analysis of health status, the relative abundance of the microbes, and diversities within and between the groups, we analysed two groups separately (i.e., Group C vs. IF) to obtain an overview of the samples from the two groups. For the LEfSe and differentially expressed gene (DEG) analyses, we used the groups to the intervention, namely, IF (*Post* and *Follow up* of Group IF) vs. NoIF (all samples from Group C and *Pre* condition of Group IF), to assess differences in the abundance of microbes specific to the treatments because these approaches detect the specific changes induced by insect feeding that should be visible even after removing the effects of daily fluctuations. We described the details of individual analyses below.

The general health condition of the subjects was evaluated by weight and faecal score. The weight was measured once during each period of the experiment (i.e., *Pre*, *Post*, and *Follow_up*) as a part of the weekly physical examination performed in our laboratory. The faecal scores were measured daily by visual inspection of the faecal shape based on three levels (partially adopted from [53]): 3 corresponded to normal faeces (solid, with little liquid), 2 corresponded to loose faeces (globules with liquid but still formed), and 1 corresponded to diarrhoea (mostly globules, a large amount of liquid, and partially muddy).

The relative abundance of the communities with normalized read counts was analysed and visualized at the phylum, family, and genus levels according to the groups (C and IF) and timing of sampling (*Pre*, *Post*, and *Follow_up*). The Shannon and Chao1 indices were calculated and statistically analysed using the vegan package (2.5–5) in RStudio software environment (ver. 1.3.1093 [54]) to evaluate the alpha diversity of the microbes in the faecal samples. The generalized linear model (GLM) was applied to the indices to determine the effects of the group and the timing of sampling, using the R function of glm in the package stats (3.6.1). Then, the degree of deviance from the model was evaluated using anova.glm function in the same package. To assess community dissimilarity (beta diversity) among and between experimental groups in microbiota and transcripts in ordination space, we conducted NMDS. Statistical differences were assessed using PERMANOVA as implemented in the R package (vegan 2.5–5) between Group C and IF. Additionally the dispersion of the beta diversities was evaluated using permutational multivariate analysis of dispersion (PERMDISP; vegan 2.5–5 package of R).

We used the LEfSe package [55] on the normalized counts at multiple taxonomic levels to conduct linear discriminant analysis (LDA) and generate a cladogram to visualize specific and significant differences in microbial abundance and taxonomic clades that emerged from the insect feeding treatment with biological consistency according to the instructions published online (https://huttenhower.sph.harvard.edu/galaxy/). For LDA, the LEfSe program was run with the threshold of 2.0 and an alpha value of 0.05 for both ANOVA (Kruskal-Wallis) and the Wilcoxon test.

The microbial communities fluctuated even in marmosets without the insect feeding treatment. Thus, DEGs were analysed using a pipeline (“EEE-baySeq”, [56]), with a false discovery rate of 5% to evaluate the changes in gene expression in the microbiota and transcripts caused by the insect feeding treatment. Our full dataset was divided according to experimental groups (C and IF) and conditions (*Pre*, *Post*, and *Follow_up*) to identify DEGs associated with increased insect consumption. Groups 1–3 (G1, G2, and G3) corresponded to the *Pre*, *Post*, and *Follow_up* conditions of Group C, while Groups 4–6 (G4, G5, and G6) corresponded to the same conditions in Group IF. Each group was compared to “others”, which included all conditions except for the group being investigated (e.g., G1 vs. others). We also performed an analysis that included comparisons among all the categories to identify any differences (named “G7”: G1 vs. G2 vs. G3 vs. G4 vs. G5 vs. G6). Thus, eight DEG patterns were obtained involving six categories (no DEG, DEG G1, DEG G2, DEG G3, DEG G4, DEG G5, DEG G6, DEG G7). Then, for the microbes and transcripts associated with any of the DEG patterns, the direction (i.e., larger or smaller than those of other categories) was determined: G1 > others, G2 > others, G3>others, G4>others, G5>others, G6>others, G1 < others, G2 < others, G3 < others, G4 < others, G5 < others, and G6 < others. For G7, only DEGs in which G5 or G6 ranked among the top or the bottom of the comparisons (i.e., “G5>G3>G4>G6>G1>G2” and “G5<G3<G4<G6<G1<G2”, for example) were considered for further analysis (G5 top, G5 bottom, G6 top, and G6 bottom). To detect the possible relationships, the microbiota and transcripts with DEGs were combinatorialy clustered in each DEG category by using the ComplexHeatmap package in RStudio. Because G1, G2, G3, and G4 were the conditions in which no insect feeding was executed, while G5 and G6 included insect feeding with different timings, any difference observed in G5 and G6 was of most interest in this study.

The functional significance of transcripts was evaluated by analysing the similarity of changes between the microbiota and transcriptome and whether these changes closely interacted with each other according to the experimental conditions. Thus, we performed a clustering analysis with Pearson’s product moment correlation coefficient among the SSU rRNA and transcriptome data with DEGs within 6 categories.

We analysed the microbiota of crickets and mealworms from the same lot as those fed to the subjects to determine whether the significant changes in the abundance of the microbial community in the samples from Group IF after insect feeding were attributable to the insects themselves that were fed to the marmosets. Whole specimens of three crickets and three mealworms were individually mashed in RNAlater, and they were further processed to extract the RNA using the same protocol as for faeces described above.

## Results

### General health condition

**Fig 1:**
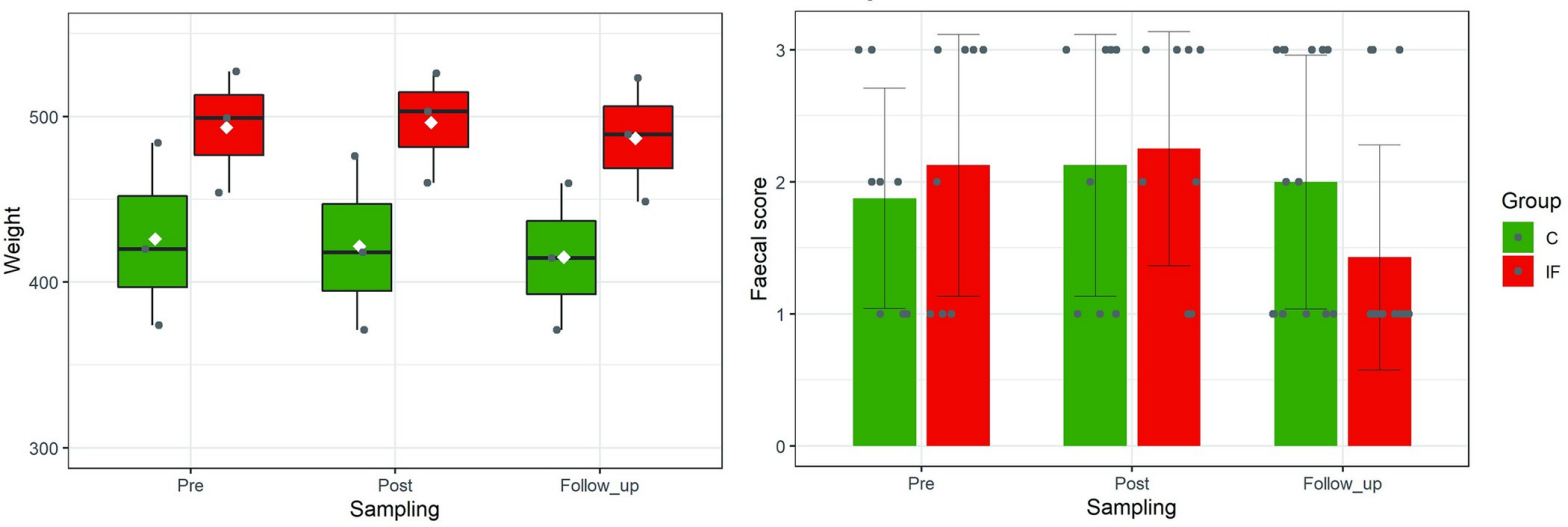
Weight (left panel) and mean faecal scores (right panel) during the experimental periods for Groups C (green) and IF (red). In the right panel, white dots and vertical bars in the box plots shows the mean and the median, respectively. In both panels, error bars show the standard deviation of the mean from three subjects. The weight was recorded individually once during each period, and the faecal scores were recorded daily.

Although the weights of Groups C and IF were significantly different according to two-way ANOVA (*F* (1, 12) = 11.78, *p* = 0.005), they were stable throughout the experimental period, as shown by the lack of significance both among the conditions (*F* (2, 12) = 0.073, *p* = 0.930) and between the interaction of the group and condition (*F* (2, 12) = 0.011, *p* = 0.999), as shown in the left panel of Fig 1. The faecal scores of the group subjected to the insect feeding treatment (Group IF) decreased (i.e., increase in the frequency of loose stools) in the *Follow_up* condition, as shown the right panel of Fig 1, but this difference was not statistically significant (*F* (2, 54) = 1.29, *p* = 0.283).

#### Microbiome composition of the marmoset gut

The total number of read counts normalized to the SSU rRNA sequences generated from the 18 faecal samples collected from the six common marmosets under the *Pre*, *Post*, and *Follow_up* conditions were 355,905.34, with an average of 19,772.52 (standard deviation (SD) 1454.93) counts per sample (see S1 Table for the normalized count data for the annotated microbes). The difference in normalized read counts per sample between Groups C and IF was not significant (*t* (16) = -0.16, *p* = 0.88, mean ± standard deviation (SD) for Group C: 65.66 ± 2.63; Group IF: 66.03 ± 6.49).

**Fig 2:**
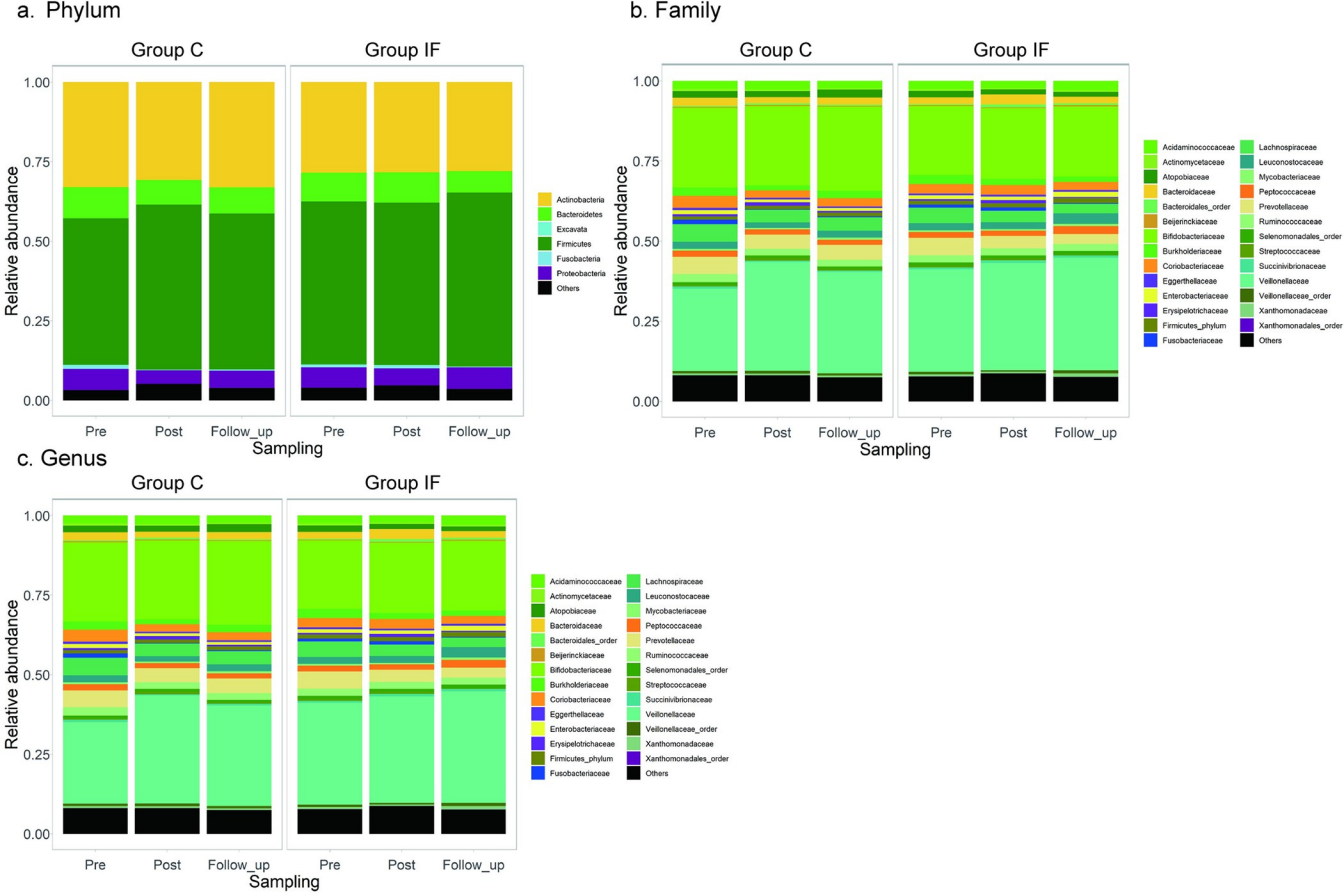
Relative abundance of microbes at the phylum (a), family (b), and genus (c) levels for Groups C and IF (left and right in each panel, respectively) under the sampling timings (*Pre*, *Post*, and *Follow_up*).

Fig 2 shows the relative abundance of the microbiota at the phylum, family, and genus levels for each group across the conditions. At each level, the taxonomic categories accounting for more than 0.5% of the total read count were listed, and those accounting for less than 0.5% and/or unable to be assigned to any categories were categorized as “others”. At the phylum level (Fig 2A), *Firmicutes*, *Actinobacteria*, *Bacteroidetes*, and *Proteobacteria* were abundant in every sample from both groups. At the family level (Fig 2B), *Veillonellaceae* (phylum *Firmicutes*) and *Bifidobacteriaceae* (phylum *Actinobacteria*) were dominant throughout the samples. By observing the same data at the genus level (Fig 2C), *Megamonas* (phylum *Firmicutes)* and *Bifidobacterium* (phylum *Actinobacteria*) were dominant under all conditions.

#### Microbiome diversity

**Fig 3:**
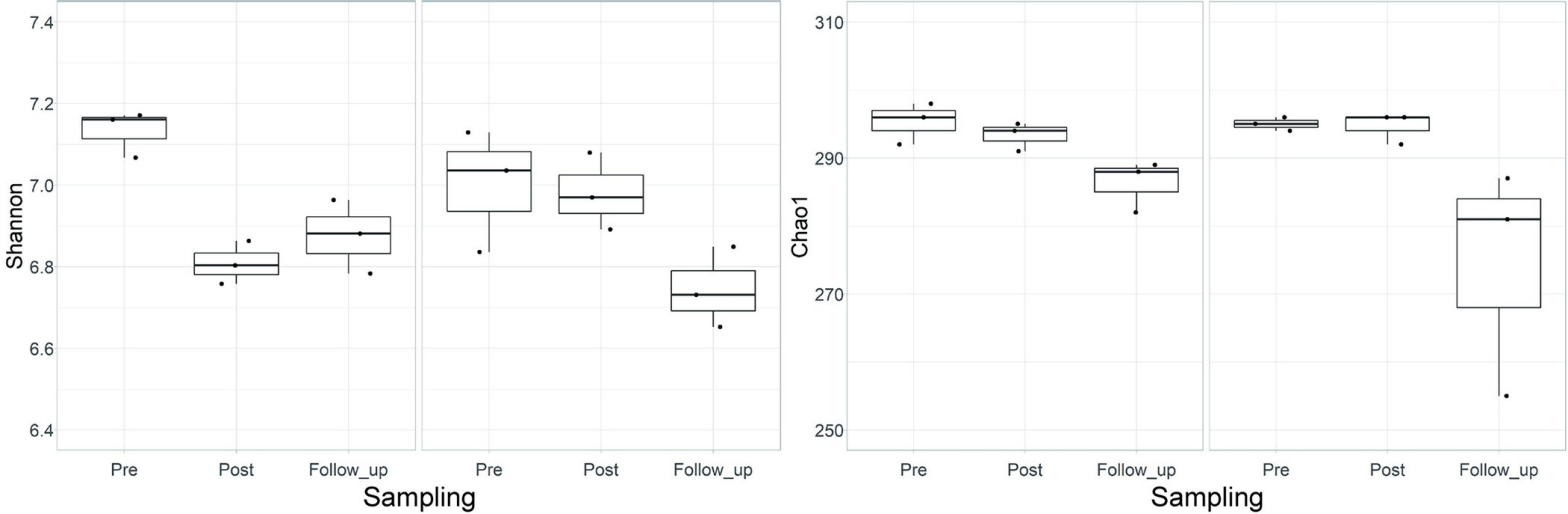
Shannon (left) and Chao1 (right) indices used to visualize the diversity within the samples of each group for the three sampling timings (left for Group C and right for Group IF in each panel, respectively). Vertical lines in the box plots show the medians.

Fig 3 shows the Shannon and Chao1 indices at each sampling time point for the groups. By applying the GLM to the Shannon index, the sampling condition (*Pre*) and the interaction with group (Group IF x *Post*) significantly affected the model (*p* = 0.006 and 0.018, respectively). The analysis of deviance in these results also revealed significant effects of the sampling time points (*Pre*, *Post*, *Follow_up*, F = 11.122, *p* = 0.002) and the interaction between sampling time points and groups (F = 5.021, *p* = 0.260), but not for the groups (F = 0.460, *p* = 0.511). On the other hand, the GLM of the Chao1 index had no significant predictors, but the deviance was significant for the sampling time point (F = 7.566, p = 0.007).

**Fig 4:**
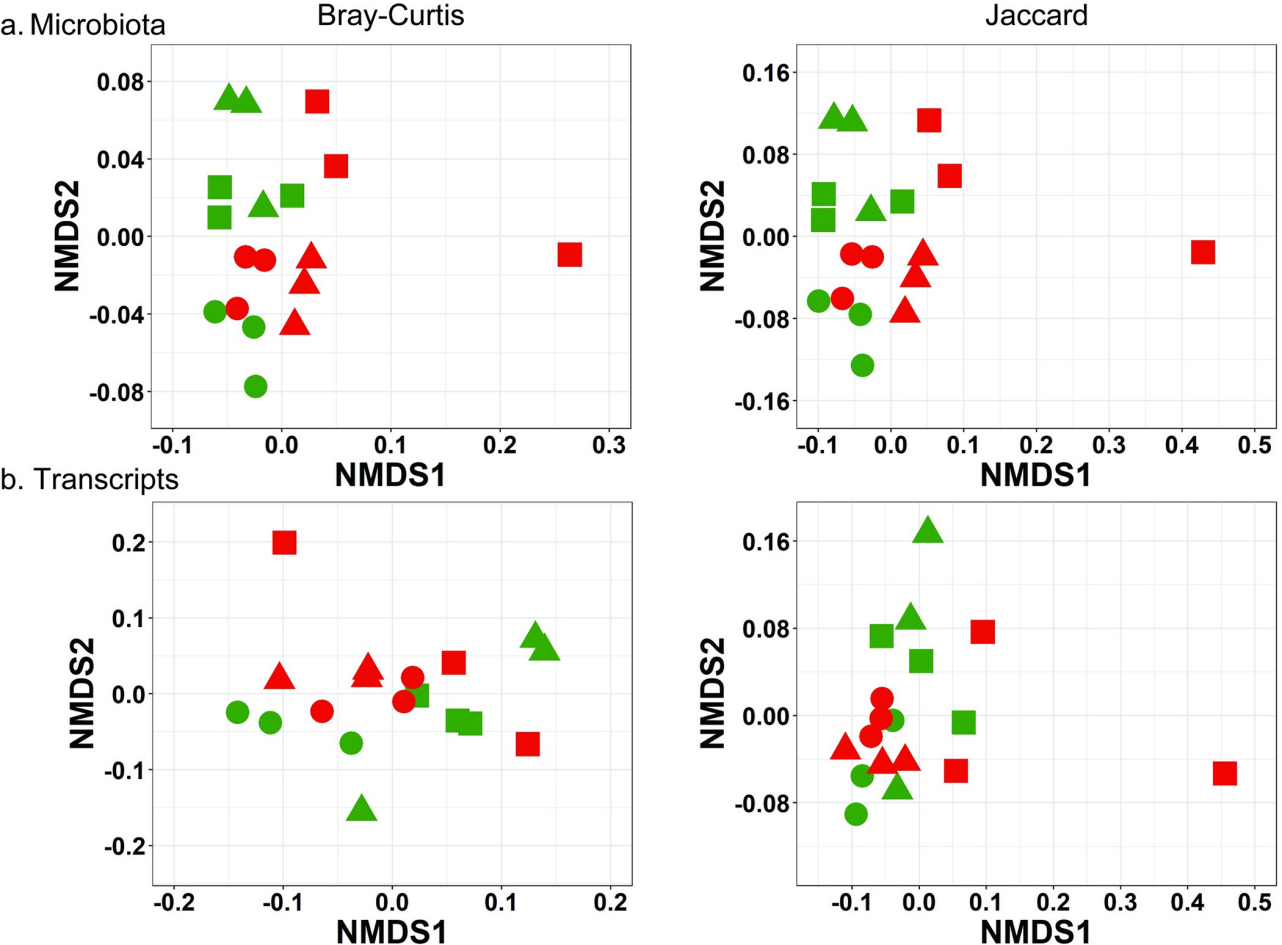
NMDS of the microbiota (a) and transcripts (b) data under the *Pre* (circle), *Post* (triangle), and *Follow_up* (square) conditions for Group C (red) and IF (blue), using Bray-Curtis (left) and Jaccard (right) indices.

Fig 4A shows the spatial visualization of NMDS of microbiota using Bray-Curtis (left panel) and Jaccard (right panel) indices. Using the Bray-Curtis index, PERMANOVA showed significant main effects of groups (F = 2.089, *p* = 0.034) and sampling time points (F = 2.585, *p* = 0.019), but not the interaction (F = 1.147, *p* = 0.350). This result was supported by the lack of significant difference in dispersion obtained using PERMDISP (F = 0.021, *p* = 0.886). Similar results were obtained for the Jaccard index (PERMANOVA: main effects of groups, F = 2.089, *p* = 0.047, sampling points, F = 2.585, p = 0.019, interaction, F = 1.147, *p* = 0.306; PERMDISP: F = 0.799, *p* = 0.385).

#### Differentially abundant gut microbes in marmosets with and without the insect feeding treatment

**Fig 5:**
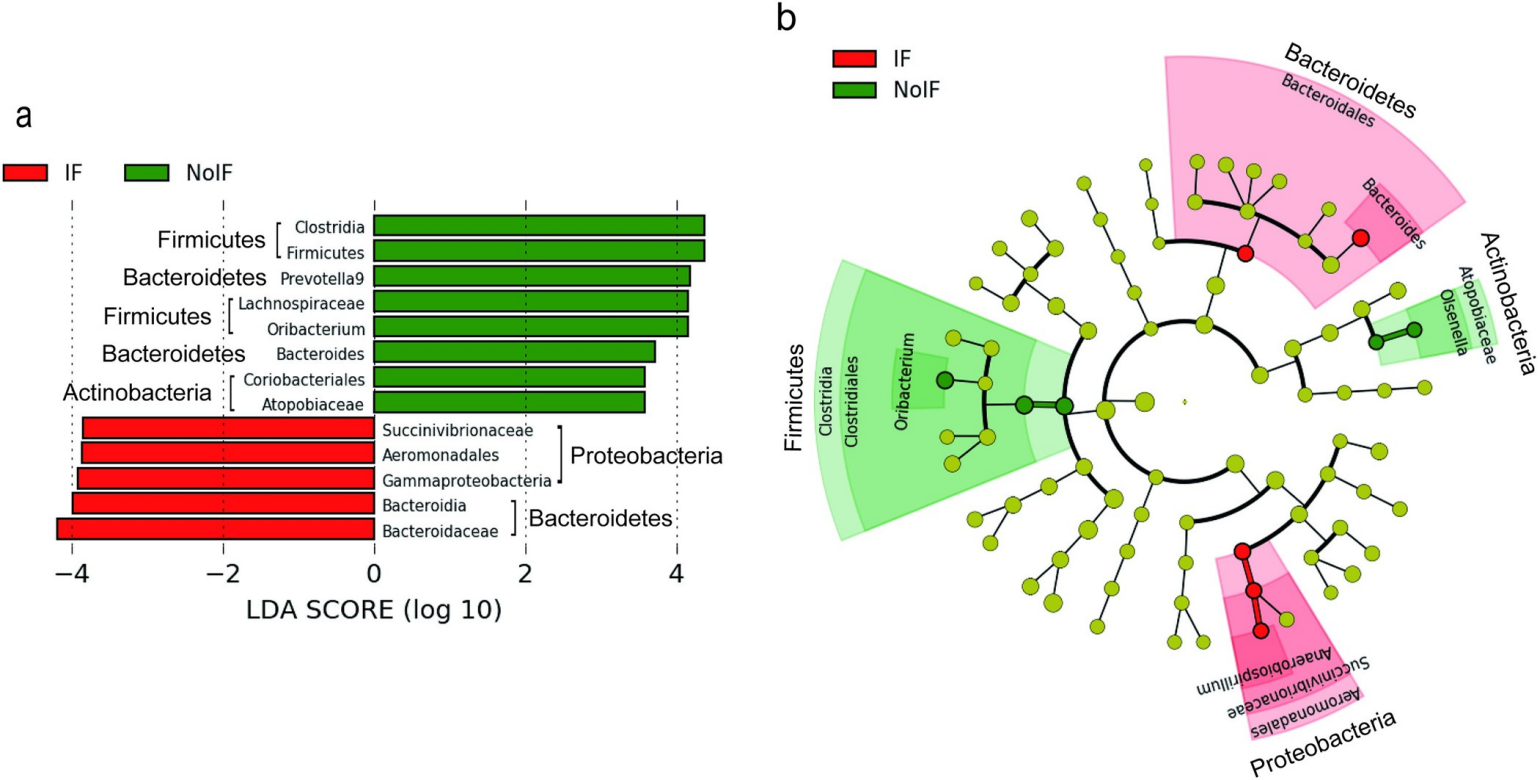
LEfSe characterization of the dominant microbial taxa according to the treatments with (IF: *Post* and *Follow_up* for Group IF) or without (NoIF: all conditions for Group C and Pre for Group IF) insect feeding. (a) LDA scores, above the threshold 2 for the microbiota on various taxonomic levels ranked according to the effect size (alpha = 0.05) for the treatments with (red, IF: *Post* and *Follow_up* for Group IF) and without (green, NoIF: all conditions for Group C and *Pre* for Group IF) insect feeding. (b) Cladogram based on the ranked list in (a) to visualize the relationships between the treatments and the phylogenetic relationships among the microbes. Phylogenetic clades are ordered from the centre of the circle, with narrower to broader taxonomic levels. Diameters of outer circles correspond to the relative abundance in the microbial community. Red and green points in the circles show the most abundant classes under the IF and NoIF treatments, respectively. Points in light green show the clades that are not significant.

Results of the LEfSe analysis are shown in Fig 5. In Group IF, differential abundance of 5 taxonomic clades (three from the phylum *Proteobacteria* and two from *Bacteroidetes*) was observed based on the LDA scores (Fig 5A). In Group NoIF, 8 taxonomic clades were differentially abundant (two from the phylum *Actinobacteria*, two from *Bacteroidetes*, and four from *Firmicutes*). The cladogram (Fig 5B) shows that abundant bacterial communities characteristic of Group IF were separatable from those characteristic of Group NoIF, as depicted by the red and green sectors without overlap. Among them, *Anaerobiospirillum* (belongs to family *Succinivibrionaceae*, phylum *Proteobacteria*) is located far away from other bacterial communities, which showed differential changes only in Group IF.

#### Differentially expressed microbial genes

**Fig 6:**
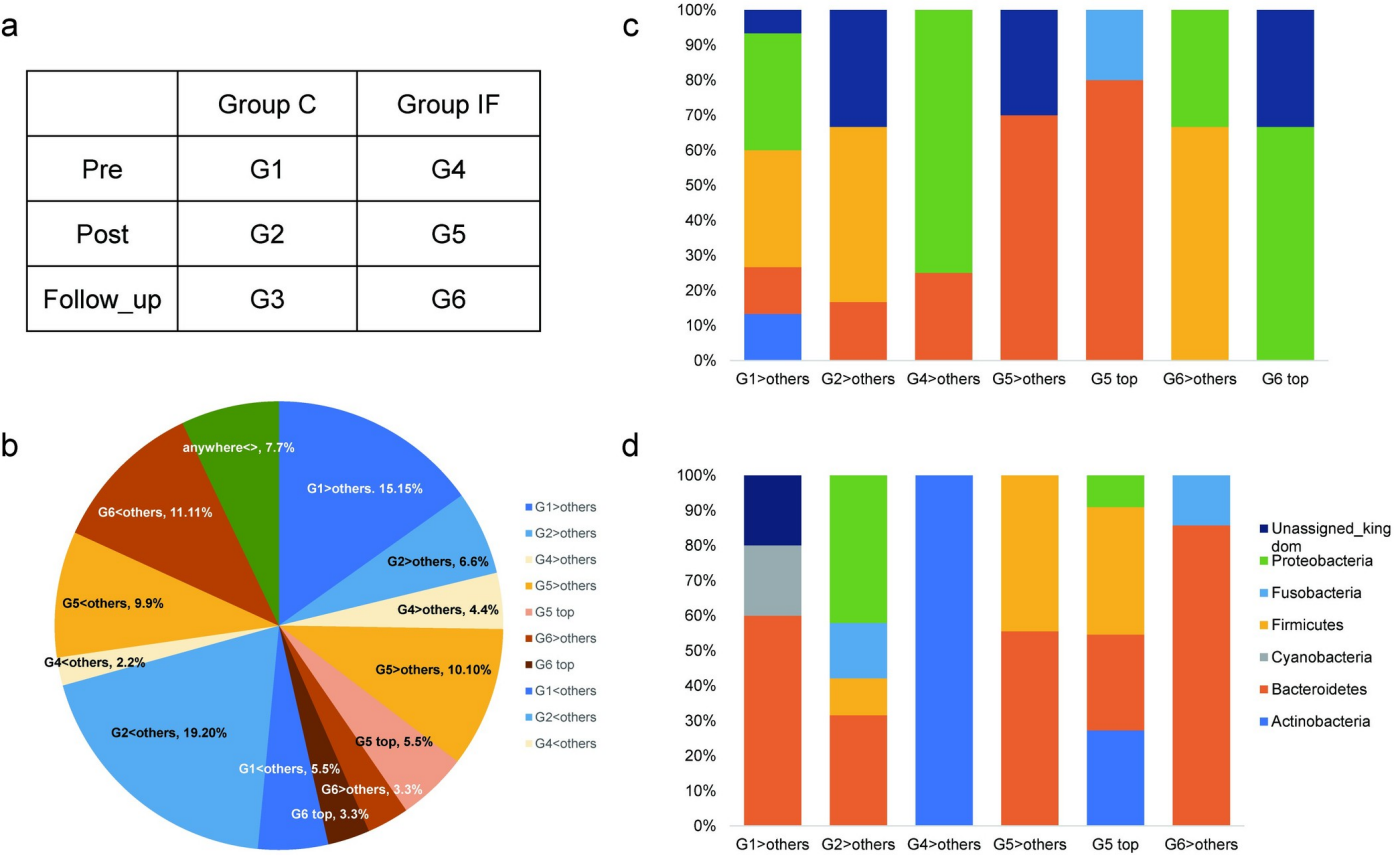
Distribution of DEG categories found in microbiota. (a) Categories of the DEG analysis. (b) Relative distribution of the DEG categories. (c) Relative distribution of microbes in each DEG category at the phylum level, showing upregulated changes. Categories under the insect-feeding treatment were G5>others, G5>anywhere, G6>others, and G6>anywhere. (d) Relative distribution of microbes in each DEG category at the phylum level, showing downregulated changes. Categories under the insect-feeding treatment were G5>others, G5>anywhere, and G6>others.

Among 300 microbes annotated by the analysis, DEGs were confirmed for 99 of them. The DEG categories and their relative distributions are presented in Fig 6A and 6B. Of 99 microbes with DEGs, a total of 21.21% were upregulated in terms of the experimental conditions (G5>others, G5 top, G6>others, G6 top), whereas a total of 20.20% were downregulated (G5<others, G6<others). No DEGs were found for the G3<>others, G5 bottom, and G6 bottom categories.

Ninety-nine microbes with DEGs were further classified into phyla. Among the upregulated phyla shown in Fig 6C, the distribution of *Bacteroidetes* was different under the *Post* condition in Group IF (i.e., G5>others and G5 top). On the other hand, among the downregulated phyla in Fig 6D, *Firmicutes* showed different distributions in the same categories.

**Fig 7:**
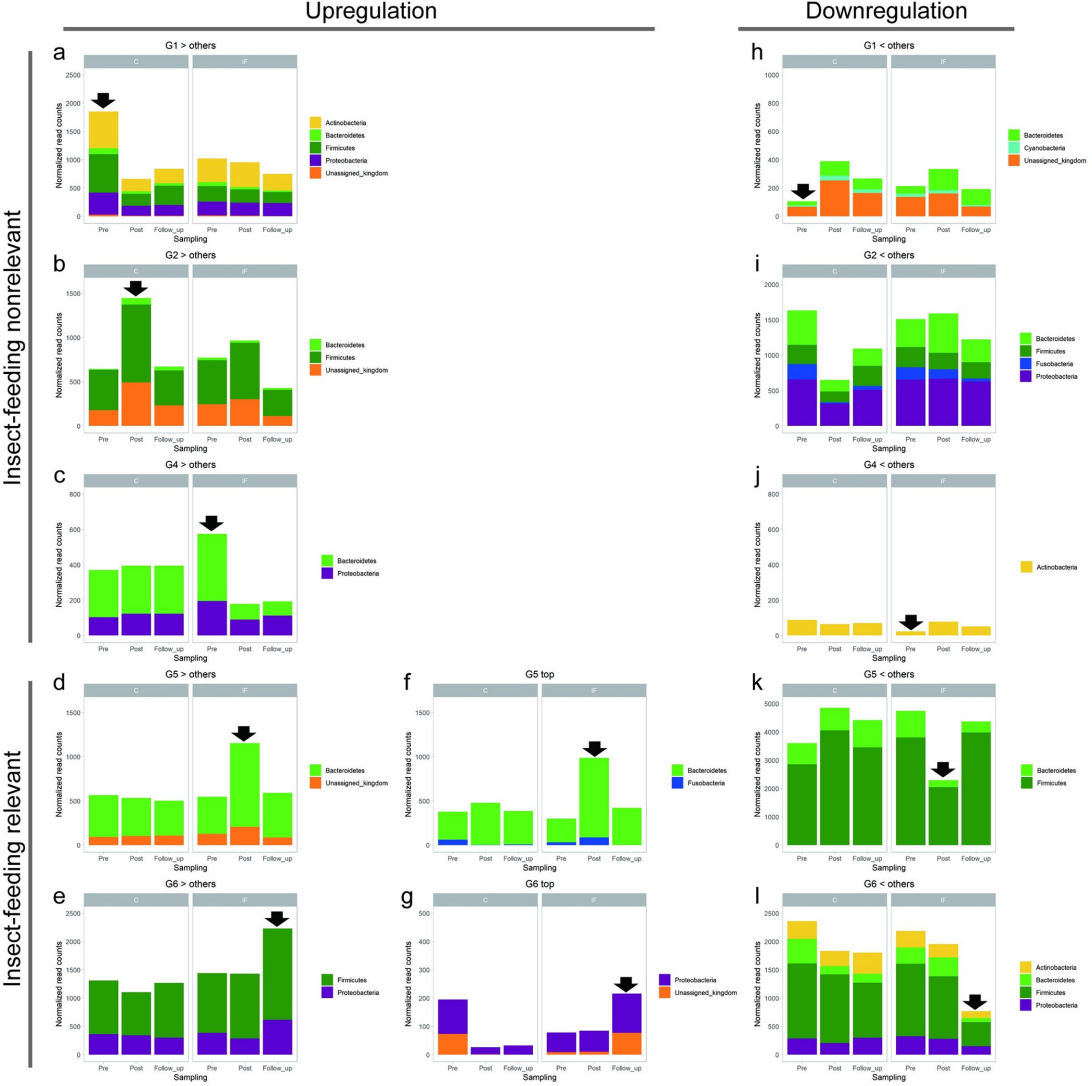
Normalized read counts of the microbes identified by the differentially expressed gene (DEG) analysis of faecal SSU rRNA at the phylum level. In each panel, the left and the right three bars show *Pre*, *Post*, *and Follow_up* conditions for Group C (left) and Group IF (right), respectively. Black arrows in the panels indicate the groups which have DEGs compared with the other groups. (a-g) Upregulated DEGs, with insect-feeding nonrelevant (a, b, c) and insect-feeding relevant (d, e, f, g) conditions. (h-l) Downregulated DEGs with insect-feeding nonrelevant (h, i, j) and insect-feeding relevant (k, l) conditions. No DEG was found under the *Follow_up* condition in Group C (G3). For f, and g, data for each microbiota were compared with those of the other groups separately. In other graphs, data for each microbiota were compared with the total of the other groups.

In Fig 7, the left two columns indicate upregulation, and the right column indicates downregulation of each DEG category at the phylum level. Under the *Post* condition in Group IF (G5), *Bacteroidetes* appeared in both upregulated (Fig 7D and 7F) and downregulated (Fig 7K) categories, whereas *Firmicutes* was only present in downregulated DEGs (Fig 7K). In the case of the *Follow_up* condition of Group IF (G6), *Actinobacteria* and *Bacteroidetes* only showed downregulated DEGs (Fig 7I), whereas *Firmicutes* and *Proteobacteria* were present in both the upregulated (Fig 7E) and downregulated (Fig 7I) categories.

**Fig 8:**
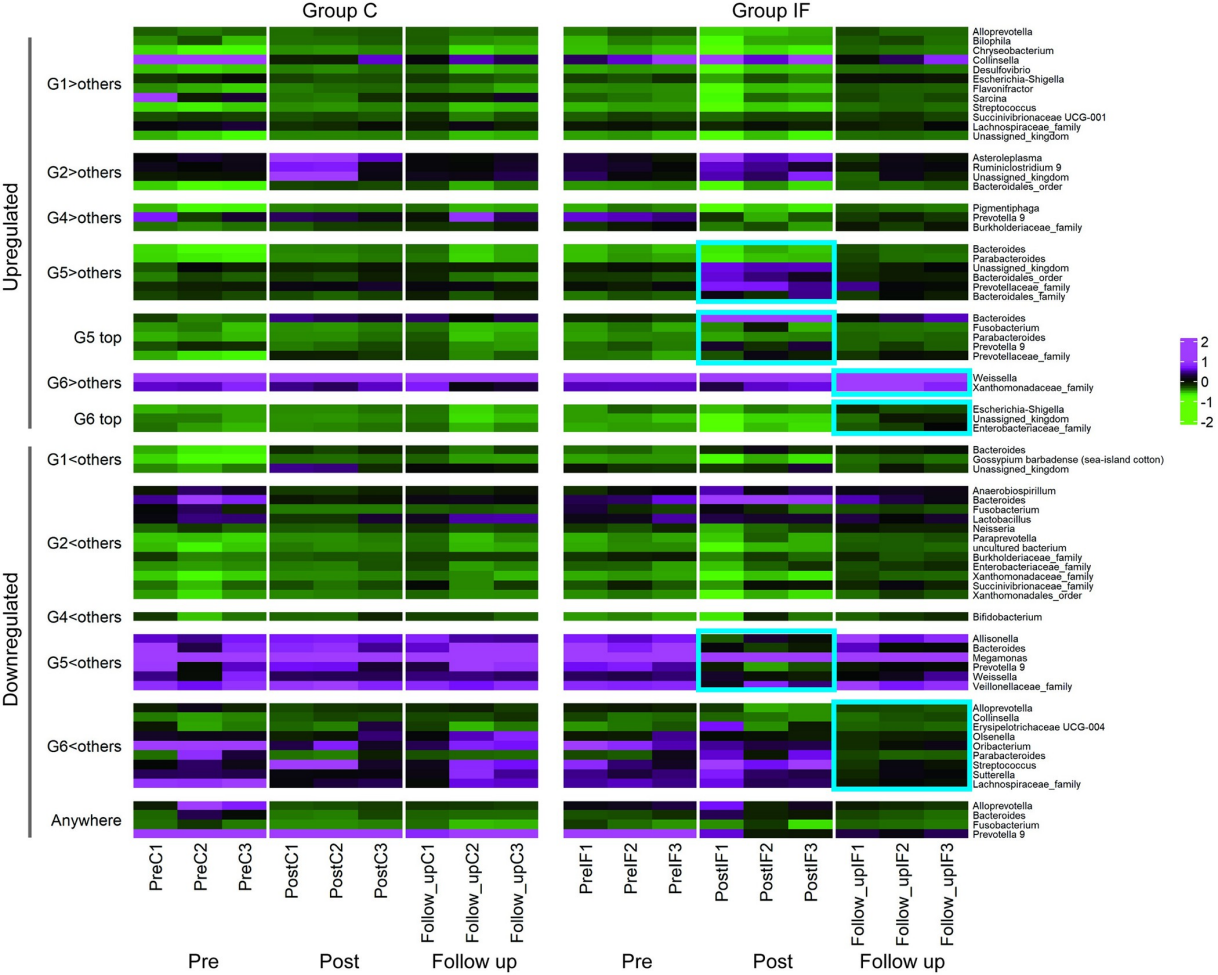
Heatmap of the SSU rRNA data (genus level) with DEGs. For microbes unable to be annotated at the genus level, upper taxonomies (i.e., order, family) were assigned. Groups C and IF are presented on left and right of the panel, respectively. For each group, the upper and lower parts present the data from upregulated and downregulated genera, respectively. G1: *Pre* condition for Group C; G2: *Post* condition for Group C; G4: *Pre* condition for Group IF, G5: *Post* condition for Group IF; G6: *Follow_up* condition for Group IF; AW: DEGs from any comparisons among the conditions. G3 is not presented because DEGs were not found under this condition (*Follow_up* condition of Group C). The heatmap with skyblue squares on the right indicates the changes in microbiota in accordance with the experimental treatments (i.e., G5 and G6 of Group IF).

In the upregulated category of the *Post* condition (G5>others, G5 top) the genera *Bacteroides*, *Parabacteroides*, *Prevotella9* (phylum *Bacteroidetes*) and *Fusobacterium* (*Fusobacteria*) were listed in the heatmap presented in Fig 8, whereas under the *Follow_up* condition (G6>others, G6 top), the genera *Weissella* (*Firmicutes*) and *Escherichia-Shigella* (*Proteobacteria*) were listed as having DEGs. In the downregulated category of the *Post* condition (G5<others), the genera *Bacteroides* (*Bacteroidetes*), *Allisonella*, *Megamonas*, and *Weissella* (*Firmicutes)* were found to have DEGs. Under the *Follow_up* condition (G6<others), the genera were *Collinsella*, *Olsenella* (*Actinobacteria*), *Alloprevotella*, *Parabacteroides* (*Bacteroidetes*), *Streptococcus*, *Erysipelotrichaceae UCG-004*, *Oribacterium* (*Firmicutes*), and *Sutterella* (*Proteobacteria*).

**Fig 9:**
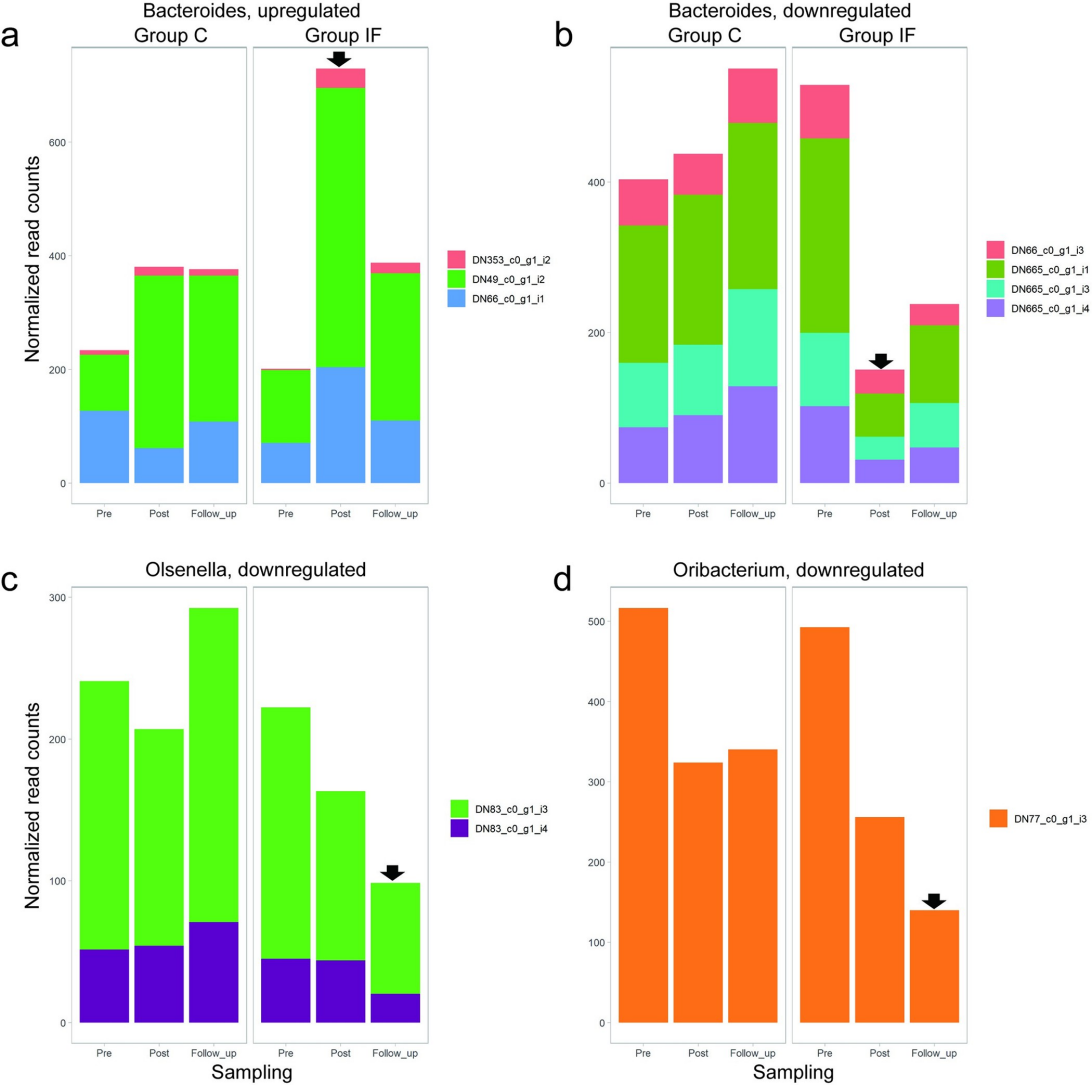
Results overlapped from the LEfSe and DEG analysis, showing the microbes specifically expressed just after the insect feeding treatment (a, b) and two weeks after the treatment (c, d), indicated by the black arrows. (a) Upregulation found under the *Post* condition in three microbes of the genus *Bacteroides*. (b) Downregulation found under the *Post* condition in four microbes of the genus *Bacteroides*. (c) Downregulation found under the *Follow_up* condition in two microbes of the genus *Olsenella*. (d) Downregulation found under the *Follow_up* condition in a microbe of the genus *Oribacterium*.

LEfSe (Fig 5) revealed some genera with differential changes relevant to the insect feeding treatment, and thus we were able to identify the specific microbes of those genera by combining the results with the DEG results presented in Fig 9 (i.e., selecting the DEGs (G5 and G6) in microbes in the genera shown to be treatment-relevant by LEfSe): *Bacteroides* (phylum *Bacteroidetes*), *Olsenella*, (phylum Actinobacteria) and *Oribacterium* (phylum *Firmicutes*). In *Bacteroides* (Fig 9A and 9B), microbes showed both upregulation and downregulation, but they were all relevant to the *Post* condition of Group IF (i.e., G5). On the other hand, the microbes in *Oribacterium* and *Olsenella* showed downregulation relevant to the *Follow_up* condition of Group IF (i.e., G6, Fig 9C and 9D, respectively).

### Transcriptomes

**Fig 10:**
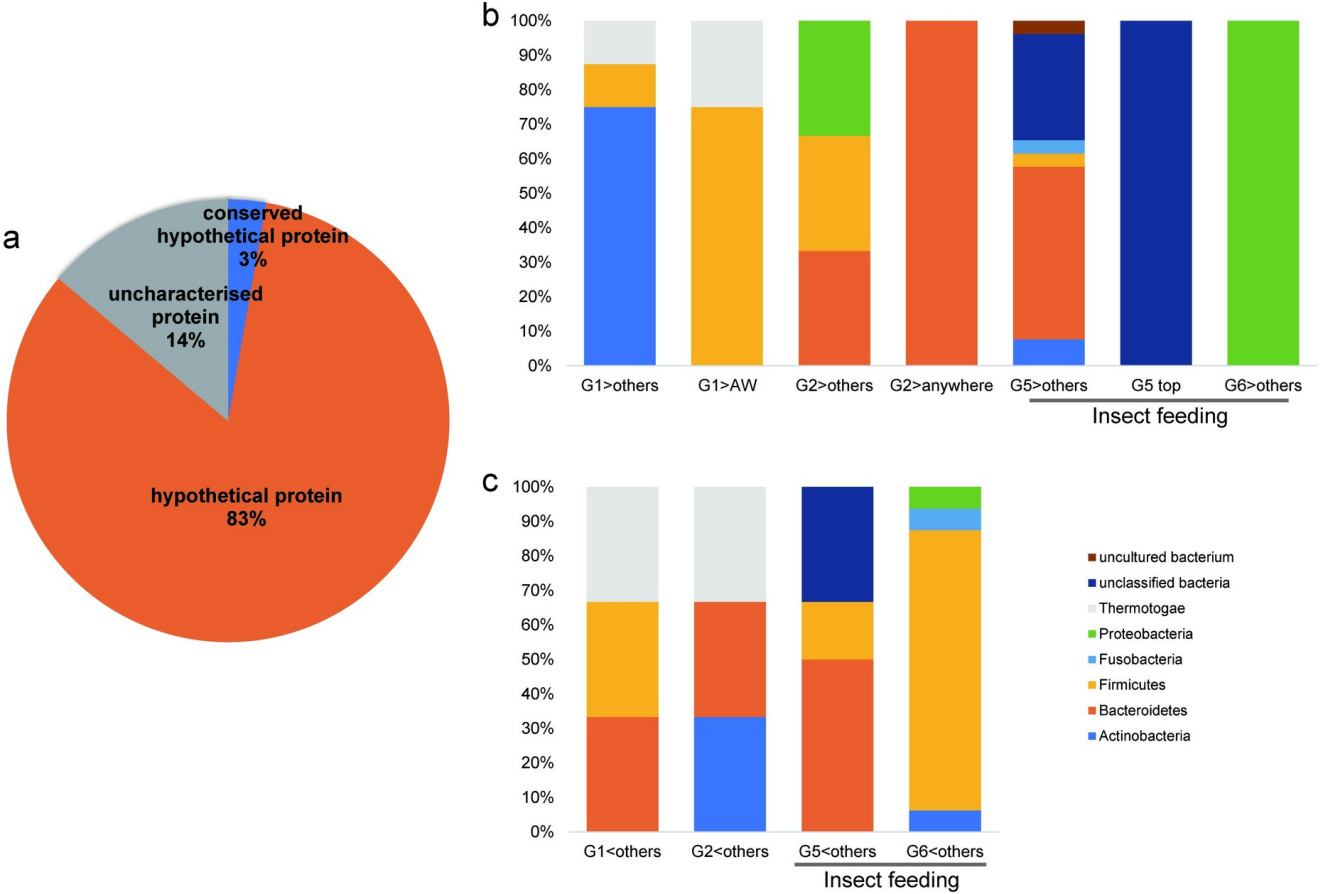
Relative distribution of DEG categories found in transcripts. (a) Relative distribution of transcript functions of DEGs. (b) Relative distribution of transcripts in each DEG category at the phylum level, showing upregulated changes. Categories under the insect-feeding treatments were G5>others, G5>anywhere, and G6>others. (c) Relative distribution of transcripts in each DEG category at the phylum level, showing downregulated changes. Categories under the insect-feeding treatment were G5>others and G6>others.

Altogether, 407 different transcript IDs were classified by BLASTP (see S2 Table for normalized count data of transcripts). Among those, 72 were analysed as DEGs, among which 83% were classified as “hypothetical proteins” from various bacteria (Fig 10A). By classifying those proteins with an e-value above 1.0e+8, it was found that transcripts originating from *Bacteroidetes* and *Firmicutes* were abundant, as shown in Fig 10B and 10C. In the case of insect feeding-relevant conditions, both *Bacteroidetes* and unclassified bacteria were abundant in both upregulated (G5>others) and downregulated (G5<others) categories. *Firmicutes* was characteristically increased in the downregulated category under the *Follow_up* condition (G6<others). *Proteobacteria* appeared only in the upregulated category under the *Follow_up* condition (G6>others).

### Relationship of the changes between the microbiota and transcriptome

**Fig 11:**
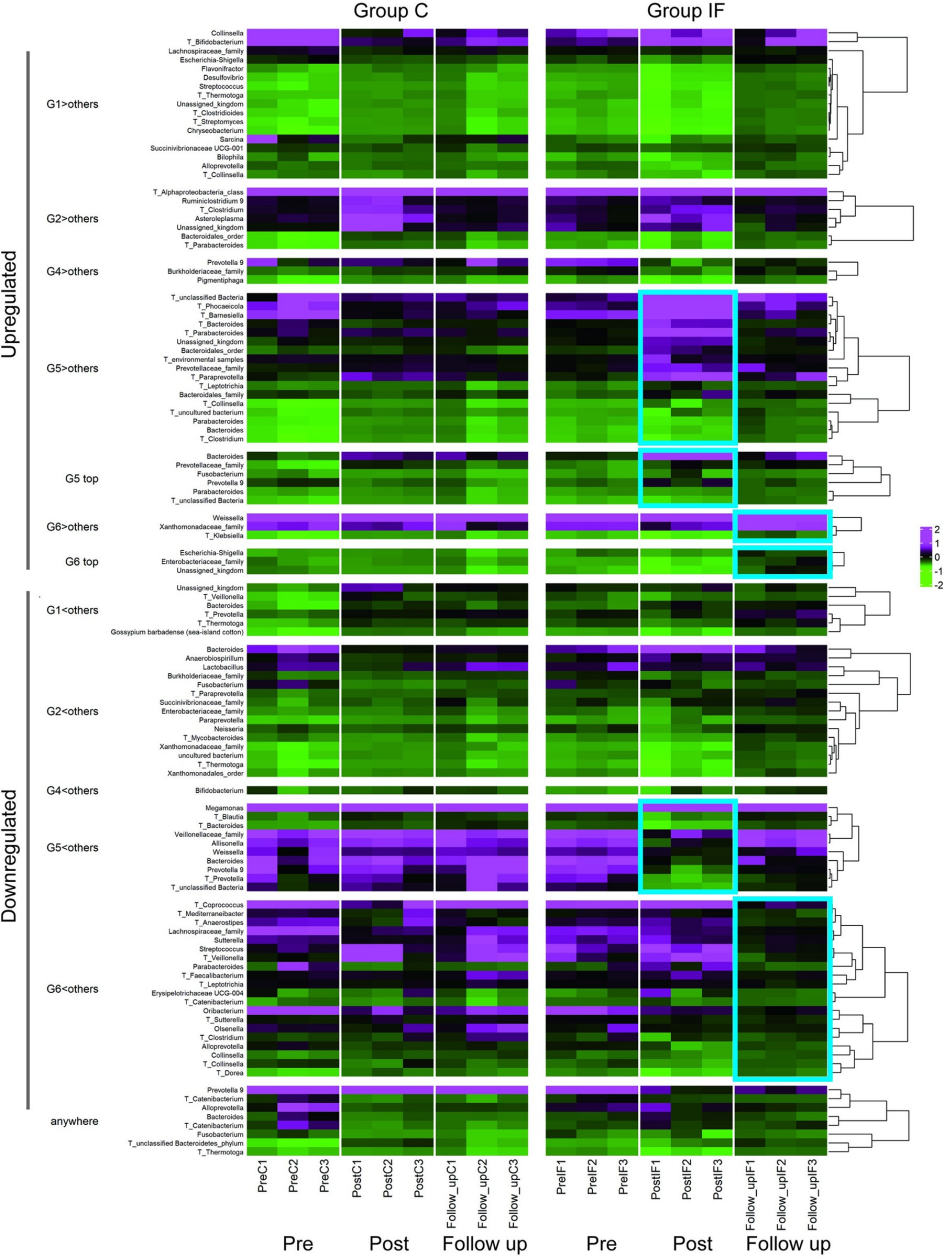
Heatmap with clustering among the SSU rRNA (genus level) and annotated transcripts with DEGs, according to the experimental period (indicated on the bottom of the heatmap) in Groups C (left) and IF (right). Transcript data are presented with T with the genera name of the originating microbes. Increases and decreases are shown in magenta and green, respectively. Hierarchical clustering for Group IF enabled items from the microbiota and transcriptome data to be listed together, where items with similar characteristics were listed close to each other. The data relevant to the experimental treatment (insect feeding) are emphasized by the skyblue squares. Upregulated and downregulated DEGs are located upper and lower panels of the figure, respectively.

Fig 11 shows the heatmap of the changes in the microbiota and transcriptome listed together in each DEG category, according to the experimental period (indicated on the bottom of the heatmap) in Groups C (left panel) and IF (right panel). The dendrogram located on the right of the heatmap shows the results of Group IF. Note that the microbiota and transcriptomes that behaved similarly were near each other in the heat map with a short clustering distance. The blue squares indicate the results relevant to the insect feeding treatment. In the case of the upregulated categories, microbes and transcripts tended to cluster separately. In the case of the downregulated categories, especially G6<others, they were more intermixed.

### Microbiota and transcripts of the insects fed to the marmosets

**Fig 12:**
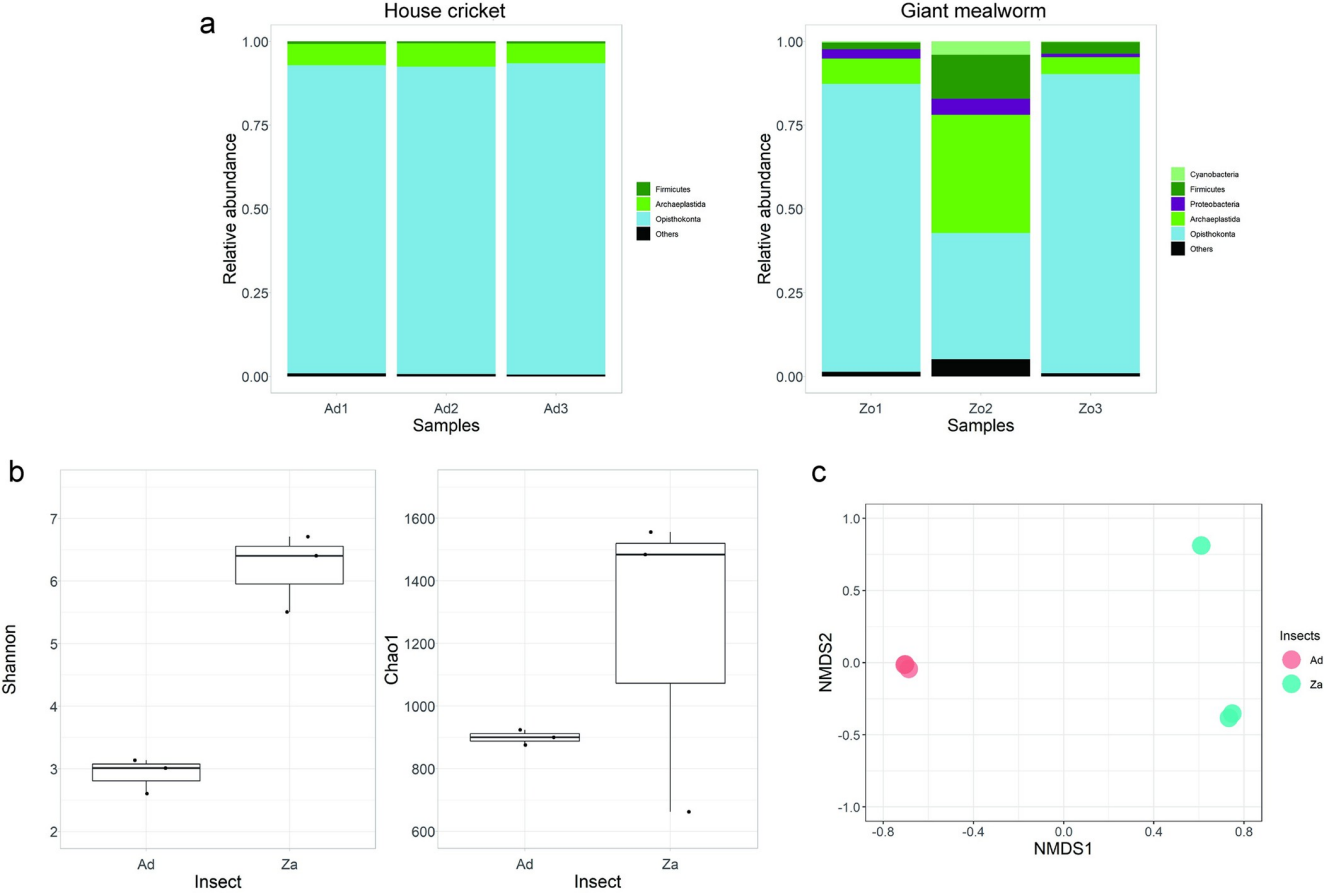
Microbial profiles of fed insects. (a) Relative abundance of microbes at the phylum level for house cricket (Ad: left) and giant mealworm (Za: right). (b) Shannon (left) and Chao1 diversity indices of the fed insects. (c) NMDS using Bray-Curtis index of crickets (Ad: pink) and worm (Za: skyblue).

Normalized count data for the microbes and transcripts of the fed insects are presented in the S3 and S4 Tables, respectively. As shown in Fig 12A, abundant microbial phyla accounting for more than 0.5% of the total reads were *Opisthokonta* (*Eukaryota*), *Archaeplastida* (*Eukaryota*), and *Firmicutes* for the crickets and *Opisthokonta*, *Archaeplastida*, *Firmicutes*, *Proteobacteria*, and *Cyanobacteria* for the meal worms. The relative abundance of the kingdom *Bacteria* was 1.09 and 7.26% of the total reads from the crickets and worms, respectively, while it was 96.38% of the total reads from the faecal samples of marmosets. All the remaining microbes were in the kingdom *Eukaryota* (98.91% and 92.04% for crickets and mealworms, respectively), which was 0.28% in the marmoset sample. Shannon and Chao1 diversity indices are presented in Fig 12B. Applying the GLM to the Shannon index, with the two insects (crickets and mealworms) and faeces of marmosets as factors, revealed statistical significance (p< 0.001). The analysis of deviance also showed the significant difference among the factors (F = 321.002, p < 0.001). Similar results were obtained for the Chao1 index (glm: p < 0.01, the analysis of deviance: F = 61.050, p < 0.001). NMDS of beta diversity, the Bray-Curtis index, is depicted in Fig 12C. Group comparison of beta diversities between marmoset microbiota and crickets or mealworms revealed a significant difference (F = 31.654, *p* = 0.001), which was accompanied by a significant difference in dispersion (F = 7.699, *p* = 0.003).

## Discussion

The present study described the characteristics of the gut microbiota of captive common marmosets by using the total RNA sequencing method, with *Firmicutes*, *Actinobacteria*, *Bacteroidetes*, and *Proteobacteria* as dominant phyla. Then, we showed that enhanced insect intake for only one week modified the microbiota population in the gut, which interacted with the transcripts simultaneously extracted from the faecal samples. Changes observed in the microbiota were not attributable to the insects themselves. More specifically, microbes in the phyla *Bacteroidetes* and *Firmicutes* showed corresponding changes in their abundance under the insect feeding treatment at the different sampling points. Some microbes in *Bacteroidetes* showed an increase or decrease upon immediate completion of the treatment (*Post* condition), followed by a decrease after two weeks (*Follow_up* condition), while some in *Firmicutes* showed a decrease under the *Post* condition, followed by an increase or decrease under the *Follow_up* condition. These results corresponded well to the changes in the abundance of transcripts having the same homologous phyla of origin. Overall, the current study indicated that a partial change in the diet for seven days had an impact on the host marmosets’ microbiota and that insect feeding naturally observed in wild populations of common marmosets has special roles.

### Microbial changes associated with insect feeding

As indicated by the LEfSe analysis (Fig 6), the phyla *Bacteroidetes* and *Proteobacteria* showed changes relevant to insect feeding, which were supported by the DEG analysis. Phylum *Firmicutes* showed a decrease and increase under the post and *Follow_up* conditions, respectively (Fig 7). These results were comparable to those obtained from the studies of other species treated with animal-concentrated diets. Studies of humans [21] and dogs [57] observed an increase in the abundance of species belonging to the genus *Bacteroides* after the consumption of animal-based diet. At the same time, the human study also reported both a decrease and increase in the abundance of some species of *Firmicutes* [21]. This study also detected an increase in the abundance of bacteria in the phylum *Proteobacteria*, including the species *Bilophila wadsworthia*, which is known to be stimulated by increased bile acid responsible for fat intake [58]. In our study, both an increase and a decrease in the abundance of *Proteobacteria* were observed under *Follow_up*, but not *Post* conditions (Fig 7E, 7G and 7I), suggesting a gradual change in bile acid metabolism in the host gut. In dogs, the abundance of *Anaerobiospirillum*, a family of the phylum *Proteobacteria*, increased and decreased after the consumption of a chicken- and beef-based diet, respectively [59], suggesting that an interactive change in the abundance of this family might occur due to the combination between specific meat and host microbiota.

The abundances at the phylum and genus levels, together with two indices of α diversity (Shannon and Chao1 indices), did not differ from each other between Groups C and IF. Insect feeding for seven days did not affect the general community of the intestinal microbiota, consistent with the results from adult humans administered 25 g of cricket powder per day for 14 days [60]. However, after focusing on the pattern of the changes corresponding to the experimental treatment by calculating the β diversity, there were significant differences between the conditions with and without insect feeding. The same patterns, no difference in α diversity but a significant difference in β diversity, were observed in a study on human subjects treated for 5 days with an animal-based diet [21]. A study examining the effects of an animal-based diet on the microbiota in dogs reported that faeces became firm and that the Shannon index increased after raw beef was added to commercial food and consumed for 14 days [57]. The reason for the lack of a significant change in α diversity indices (Shannon and Chao1) after insect feeding in the current study would partially be attributable to unexpected fluctuations observed in the samples of Group C. Thus, evaluation of the stability of the microbial community would be necessary to compare the effects of short-term intervention (seven days) by taking additional samples in the *Pre* period, for example.

### Limitations and future perspectives of the study

Our analysis using total RNA-seq, which could concurrently detect the dynamics of the microbiota and transcripts, was effective in searching for functional genes that are currently unidentifiable after establishing a metagenomic database of the transcriptome with a full-length cDNA library. However, we were unable to detect many functional proteins using our RNA-seq methods. The results presented here were the best to ensure the full reliability of identification of the genes. The functional significance of the transcriptomes was only able to be inferred by the microbiota that showed similar changes across the experimental conditions. A metagenome analysis, annotation, and identification of hits in the genome in an additional set of analysis might improve our protocol to capture more functional proteins. Then, we will obtain a wider view that would integrate the changes in microbiota, transcripts, and host responses to understand the effect of insect feeding, as the subjects have a long history of insect foraging.

In the present study, we used frozen crickets and giant mealworms instead of live mealworms. The results might have been different from those obtained in the study using live insects. The dose of insects fed to marmosets may have been too small to exert drastic effects on their microbiota and transcripts. We determined the dose because we wanted to ensure that they completely ate all of the insects; therefore, there was little difference in doses fed to every animal without malnutrition caused by the change in diets. Nevertheless, the present study showed that feeding common marmoset insects changes their physiological status by balancing the microbiota to modulate metabolites. Consideration must be given to how much and what types of insects we should feed captive marmosets because there is a risk of overeating in the breeding cages but not in bushes in the wild. For example, some insect larvae are rich in fat and lack calcium, and it is therefore recommended to feed the insects a high calcium diet before feeding the insects to marmosets [10]. Insects with a high phosphorous-calcium rate should not be provided in abundance to prevent the malabsorption of calcium [32]. Thus, further studies are clearly needed to determine the long-term effect of insect feeding in captive animals and what types of insects are most beneficial for their health while simultaneously monitoring the changes in the faecal microbiota and transcriptome.

## Conclusion

The present study showed that adding insects to the regular food regimen for seven days could have a distinct effect on the microbiota and transcripts of captive common marmosets. The total RNA-seq method was used to analyse the microbiota and transcripts simultaneously, and the correlational analysis suggested that they did interact with each other. Thus, enhanced insect feeding could activate the physiological dynamics that have been evolutionarily developed in this species in wild habitats. The obtained results help us to understand the interaction between the host and the microbiota via food sources and suggest that the feeding ecology in wild habitats is an important key to developing food regimens appropriate for the microbiota of common marmosets.

## Supporting information

S1 Table Normalized counts of microbes annotated by QIIME 2 vsearch for Groups C and IF. (XLSX)

S2 Table Normalized counts of transcripts annotated by blastp for Groups C and IF. (XLSX)

S3 Table Normalized counts of the microbes of the insects (house crickets and giant mealworm). (XLSX)

S4 Table Normalized counts of the transcripts of the insects. (XLSX)

## List of abbreviations

ANOVA: analysis of variance
Group C: control group
Group IF: insect feeding group
LDA: linear discriminant analysis
LEfSe: linear discriminant analysis effect size
MWS: marmoset wasting syndrome
ORF: open reading frame
PERMANOVA: permutational multivariate analysis of variance
PERMDISP: permutational multivariate analysis of dispersion
SSU rRNA: small subunit ribosomal ribonucleic acid
total RNA-seq: total RNA sequencing
WMS: wasting marmoset syndrome
